# Kentucky State Medical Society

**Published:** 1877-05

**Authors:** 


KENTUCKY STATE MEDICAL SOCIETY.—The annual meeting of this Society was held at Louisville, on the 3d of April. Dr. R. W. Gaines, of Hopkinsville, President, in the chair. The following officers were elected for the ensuing year. President, Dr. L. P. Yandell, Jr., of Louisville; Vice Presidents, Drs. J. Dismukes, of Mayfield, and W. B. Rodman, of Frankfort. Frankfort was chosen as the place of meeting in 1878.

Dr. L. S. McMurtry, of Danville, reported that the M'Dowell Monument Fund amounted to $434. Subscriptions were given on the spot, increasing the fund to $1,054.

Dr. Baker, of Shelbyville, offered the following resolutions, which were adopted unanimously:—

*Resolved,* That this Society is in full accord with the American Medical College Convention, seeking to elevate the standard of medical education in this country.

*Resolved,* That summer schools, which enable students to graduate after from eight to nine months study, are exerting an evil influence upon the profession.

*Resolved,* That a winter and summer course by the same school, and graduation at the end of each, tends to deteriorate the standing of the medical profession.

